# A New Chaotic System with Multiple Attractors: Dynamic Analysis, Circuit Realization and S-Box Design

**DOI:** 10.3390/e20010012

**Published:** 2017-12-27

**Authors:** Qiang Lai, Akif Akgul, Chunbiao Li, Guanghui Xu, Ünal Çavuşoğlu

**Affiliations:** 1 School of Electrical and Automation Engineering, East China Jiaotong University, Nanchang 330013, China; 2 Department of Electrical and Electronics Engineering, Faculty of Technology, Sakarya University, Serdivan 54187, Turkey; 3 School of Electronic and Information Engineering, Nanjing University of Information Science and Technology, Nanjing 210044, China; 4 School of Electrical and Electronic Engineering, Hubei University of Technology, Wuhan 430068, China; 5 Department of Computer Engineering, Faculty of Computer and Information Sciences, Sakarya University, Serdivan 54187, Turkey

**Keywords:** new chaotic system, multiple attractors, electronic circuit realization, S-Box algorithm

## Abstract

This paper reports about a novel three-dimensional chaotic system with three nonlinearities. The system has one stable equilibrium, two stable equilibria and one saddle node, two saddle foci and one saddle node for different parameters. One salient feature of this novel system is its multiple attractors caused by different initial values. With the change of parameters, the system experiences mono-stability, bi-stability, mono-periodicity, bi-periodicity, one strange attractor, and two coexisting strange attractors. The complex dynamic behaviors of the system are revealed by analyzing the corresponding equilibria and using the numerical simulation method. In addition, an electronic circuit is given for implementing the chaotic attractors of the system. Using the new chaotic system, an S-Box is developed for cryptographic operations. Moreover, we test the performance of this produced S-Box and compare it to the existing S-Box studies.

## 1. Introduction

The discovery of the well-known Lorenz attractor [1] in 1963 opened the upsurge of chaos research. In the decades thereafter, a large number of meaningful achievements on chaos control, chaotification, synchronization and chaos application have emerged continuously. Great changes have also been made to the understanding of chaos. Scholars began to think more about a way to produce chaos rather than blindly suppress chaos. The generation of chaotic attractors in three-dimensional autonomous ordinary differential systems has been of particular interest. As we all know, a multitude of typical systems with chaotic attractors were found, including Rössler system, Chen system, Sprott system, Lü system, etc. [2,3,4,5,6,7,8].

With the further research of chaos, scientists found that some nonlinear dynamic systems not only have a chaotic attractor but also coexist with multiple attractors for a set of fixed parameter values. The coexisting attractors may be fixed points, limit cycles, strange attractors, etc. The number and type of attractors are usually associated with parameters and initial conditions of the system. Each attractor has its own basin of attraction which is composed of the initial conditions leading to long-term behavior that settles onto the attractor. The phenomenon of multiple attractors can be seen in many biological systems and physical systems [9,10,11]. In recent years, the low-dimensional autonomous chaotic systems with multiple attractors have aroused scholars’ research enthusiasm. Li and Sprott found multiple attractors in chaotic systems by numerical analysis and introduced the offset boosting method and conditional symmetry method for producing multiple attractors in differential systems [12,13,14,15,16]. Kengne et al. analyzed the multiple attractors of simple chaotic circuits, which can be described by differential equations [17,18]. Bao and Xu put forward some memristor-based circuit systems with multiple chaotic attractors [19,20]. Lai et al. proposed some three-dimensional and four-dimensional continuous chaotic systems with multiple attractors [21,22,23]. Wei et al. attempted to reveal the intrinsic mechanism of the multiple attractors by analyzing the bifurcation of the system [24]. The investigation of chaos and multiple coexisting attractors is indeed a very interesting research issue in academia. It helps to recognize the dynamic evolution of the actual system and promote the study of complexity science.

Chaotic systems have been found to be used in many areas. The most valuable application is cryptology. Chaotic system, in view of its rich dynamic behaviors and initial sensitivity, provides the mixing and spreading properties, which are the general requirements of encryption [25,26]. The S-Box is known as the most basic unit with scrambling function in block encryption algorithms. A good S-Box can make the encryption algorithm have higher security and better ability to withstand attacks. Although there have been many works on chaotic S-Box design, it is still important to generate S-Box according to some unique chaotic systems. Before applying the chaotic system to engineering fields, it is necessary to realize it through electronic circuits in order to prove its real existence. Based on circuit theory and simple circuit elements, chaotic signals can be generated in oscilloscopes. So far, the electronic circuit has become an important tool for the analysis of chaotic systems [27,28,29,30].

This present paper considers a special polynomial chaotic system with the following features: (i) it has three nonlinearities xz,yz,xyz and the invariance of transformation (x,y,z)↦(−x,−y,z); (ii) it performs a butterfly attractor; and (iii) it has a stable equilibrium, an unstable equilibrium and two stable equilibria, three unstable equilibria for different parameter conditions, and experiences mono-stability, bi-stability, mono-periodicity, bi-periodicity, one strange attractor and two coexisting strange attractors. After investigating the dynamic behavior of the system, an electronic circuit and an S-Box are designed according to the system.

The paper is arranged as follows: Section 2 describes the chaotic system and shows its butterfly attractor. Section 3 analyzes the stability of the equilibria. Section 4 studies the dynamic behavior of the system. Section 5 considers the electronic circuit realization of the system. Section 6 establishes the S-Box according to the system, and Section 7 summarizes the conclusions of this paper.

## 2. The Description of a Chaotic System

**Figure 1:**
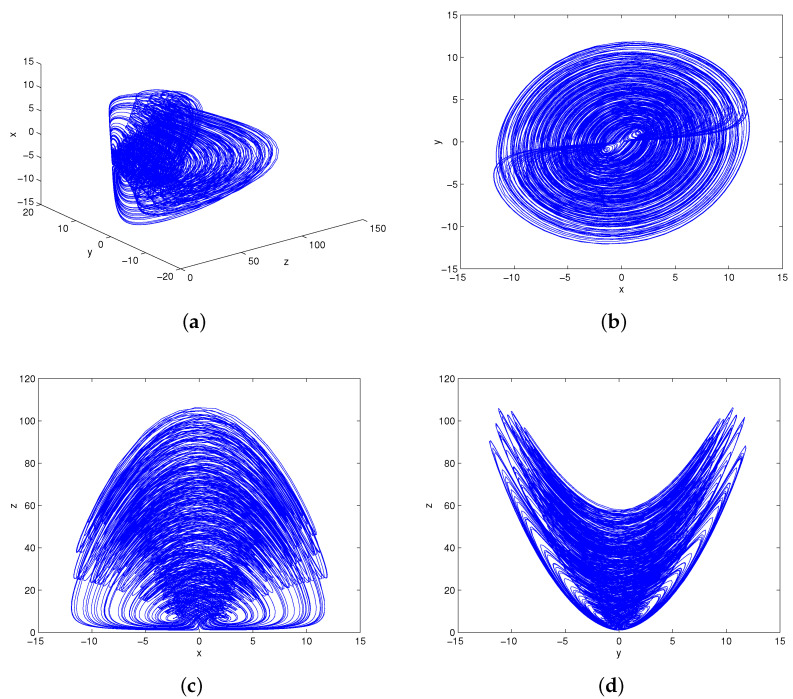
The butterfly attractor of system (1): (**a**) x−y−z; (**b**) x−y; (**c**) x−z; (**d**) y−z.

**Figure 2:**
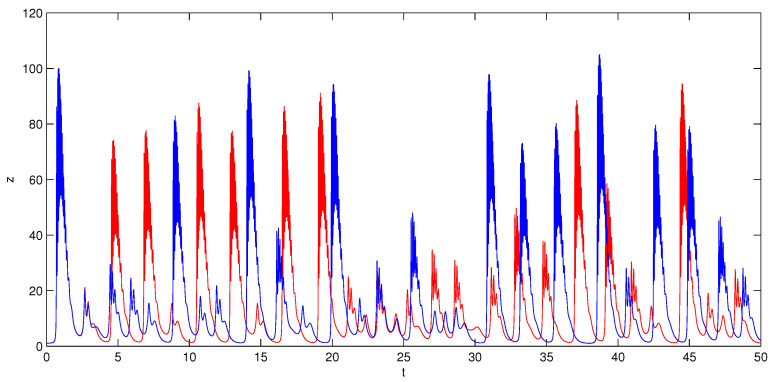
The time series of variable *z* generated from initial conditions (1,1,1) (red color) and (1,1,1.001) (blue color).

**Figure 3:**
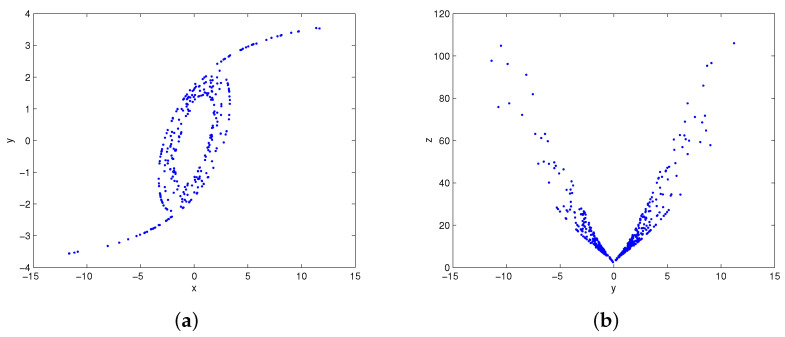
The Poincaré maps of system (1) with crossing sections: (**a**) $\Delta_{1}$; (**b**) $\Delta_{2}$.

The chaotic system proposed in this paper can be expressed as the following set of differential equations:

(1) $$\left\{\begin{array}{c} \dot{x}=ax-yz,\\ \dot{y}=-by+xz,\\ \dot{z}=-cz+xyz+k, \end{array}\right.$$

with state vector $\left(x,y,z\right)\in R^{3}$ and parameter vector $\left(a,b,c,k\right)\in R^{4}$. A butterfly attractor can be observed by numerical simulation on Matlab software (Matlab 8.0, MathWorks, Natick, MA, USA). The phase portraits of system (1) under parameters (a,b,c,k)=(4,9,4,4) and initial condition (1,1,1) are shown in Figure 1. It visually demonstrates that system (1) displays an attractor as the system trajectories will eventually move to a bounded region. The Lyapunov exponents of the system are calculated as $l_{1}=1.7729$, $l_{2}\;=\;0.0000$, $l_{3}=-7.5949$. The Lyapunov dimension is $D_{l}=2-l_{1}/l_{3}=2.2334$, so it can be determined that the attractor is a chaotic attractor. The time series of *z* generated from two very close initial conditions (1,1,1) and (1,1,1.001) are plotted in Figure 2. At the beginning, they are almost the same, but their differences are increasing after a number of iterations. That is to say, system (1) is sensitive dependence on initial conditions and its future behavior is unpredictable in the long term. The Poincaré map of system (1) is obtained via selecting the sections $\Delta_{1}=\{\left(x,y\right)\in R^{2}\left|z=10\}\right.$ and $\Delta_{2}=\{\left(y,z\right)\in R^{2}\left|x=0\}\right.$. As shown in Figure 3, the Poincaré map is a sheet of point set. It is consistent with the nature of chaos.

## 3. The Stability of Equilibria

Suppose that parameters a,b,c,k are all positive real numbers. The equilibria of system (1) can be obtained by solving $\dot{x}=\dot{y}=\dot{z}=0$. If $k\geq c\sqrt{ab}$, system (1) has only one equilibrium O(0,0,k/c). If $k<c\sqrt{ab}$, system (1) has three equilibria as follows:

$$\begin{array}{c} \begin{array}{c} \;O(0,0,k/c),\\ \;O_{1}\left(\sqrt{(c\sqrt{ab}-k)/a},\sqrt{(c\sqrt{ab}-k)/b},\sqrt{ab}\right),\\ O_{2}\left(-\sqrt{(c\sqrt{ab}-k)/a},-\sqrt{(c\sqrt{ab}-k)/b},\sqrt{ab}\right). \end{array} \end{array}$$

**Proposition** **1.** *Suppose that b>a>0,k>0, and the parameter c satisfies the following condition:*

(2) $$\frac{k}{\sqrt{ab}}<c<\frac{2k[\left(a^{2}+b^{2}\right)\sqrt{ab}+k\left(b-a\right)]}{\sqrt{ab}\left[{\left(a+b\right)}^{2}\sqrt{ab}+k\left(b-a\right)\right]},$$

*then the equilibria $O_{1}$ and $O_{2}$ of system (1) are asymptotically stable.*

**Proof.**  By linearizing the system (1) at the equilibrium, the Jacobian matrix is given by

(3) $$H=\left(\begin{array}{ccc} a\;\; & -z\;\; & -y\\ z\;\; & -b\;\; & x\\ yz\;\; & xz\;\; & xy-c \end{array}\right).$$

By using $\left|\lambda I-H\right|=0$, the corresponding characteristic equation evaluated at $O_{1}$, $O_{2}$ is obtained as

(4) $$\lambda^{3}+w_{1}\lambda^{2}+w_{2}\lambda +w_{3}=0,$$

where

$$\begin{array}{c} \begin{array}{c} \;w_{1}=\left(b-a+\frac{k}{\sqrt{ab}}\right),\\ \;w_{2}=\left(a-b\right)\left(c-\frac{2k}{\sqrt{ab}}\right),\\ w_{3}=4ab\left(c-\frac{k}{\sqrt{ab}}\right). \end{array} \end{array}$$

 ☐

According to the Routh–Hurwitz criterion, the equilibria $O_{1}$, $O_{2}$ are stable if all the roots of Equation (4) have negative real parts. This requires that $w_{1}>0$, $w_{2}>0$, $w_{3}>0$ and $w_{1}w_{2}>w_{3}$. It is easy to verify that $w_{1}>0$, $w_{2}>0$, $w_{3}>0$ if b>a>0,k>0 and

(5) $$\frac{k}{\sqrt{ab}}<c<\frac{2k}{\sqrt{ab}}.$$

To make $w_{1}w_{2}>w_{3}$, the parameter *c* should meet

(6) $$c<c_{0}=\frac{2k[\left(a^{2}+b^{2}\right)\sqrt{ab}+k\left(b-a\right)]}{\sqrt{ab}\left[{\left(a+b\right)}^{2}\sqrt{ab}+k\left(b-a\right)\right]}.$$

Since $c_{0}<\frac{2k}{\sqrt{ab}}$, then $O_{1}$, $O_{2}$ are asymptotically stable if b>a>0,k>0, $\frac{k}{\sqrt{ab}}<c<c_{0}$. When the parameter *c* passes through the critical value $c_{0}$, then double Hopf bifurcation occur with two limit cycles branched from $O_{1}$, $O_{2}$ and system (1) loses its stability.

**Proposition** **2.** Suppose that b>a>0,c>0,k>0, then: (i) the equilibrium O is unstable for $c>\frac{k}{\sqrt{ab}}$; and (ii) the equilibrium O is asymptotically stable for $c\leq \frac{k}{\sqrt{ab}}$.

**Proof.**  The characteristic equation evaluated at *O* is given by

(7) $$\left(\lambda +c\right)[c^{2}\lambda^{2}+\left(b-a\right)c^{2}\lambda +k^{2}-abc^{2}]=0.$$

If $c>\frac{k}{\sqrt{ab}}$, then Equation (7) has a root with a positive real part. Thus, *O* is unstable. If $c<\frac{k}{\sqrt{ab}}$, all the roots of Equation (7) have negative real parts, which implies that *O* is stable. When $c=\frac{k}{\sqrt{ab}}$, Equation (7) has three roots $\lambda_{1}=0,\lambda_{2}=a-b,\lambda_{3}=-c$. Therefore, *O* is non-hyperbolic equilibrium. It can be verified that *O* is asymptotically stable by applying the center manifold theorem. ☐

## 4. The Evolution of Multiple Attractors

Detailed investigation of the complex dynamic behaviors of system (1) is presented in this section. Simulation experiments including bifurcation diagrams, phase portraits, Lyapunov exponents, and Poincaré maps give a close and intuitive look at system (1). There is a wealth of chaotic dynamics associated with the fractal properties of the attractor in system (1). With the change of parameters, system (1) experiences stable state, periodic state and chaotic state. For different initial values, system (1) performs different types of attractors with independent domains of attraction.

### 4.1. Dynamic Evolution with Parameter c

**Figure 4:**
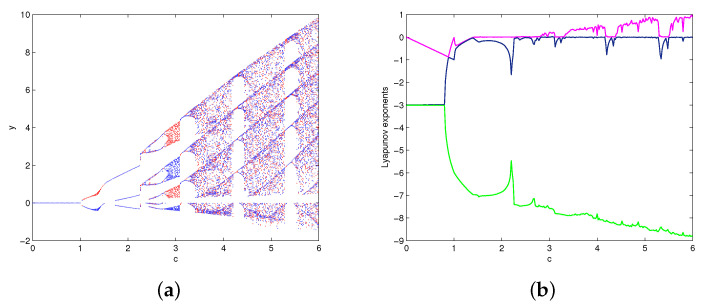
The bifurcation diagrams (**a**) and Lyapunov exponents (**b**) of system (1) versus c∈(0,6).

**Figure 5:**
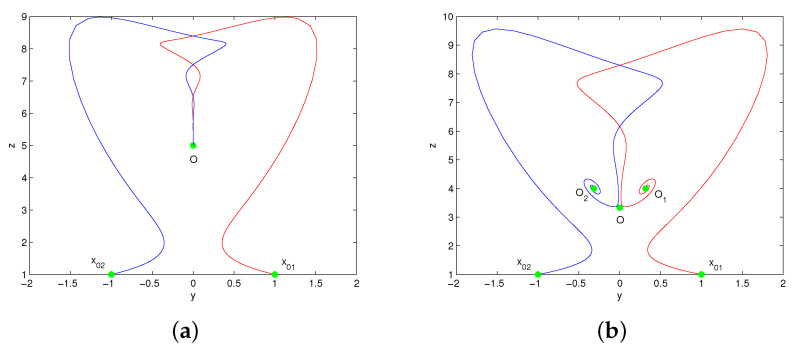

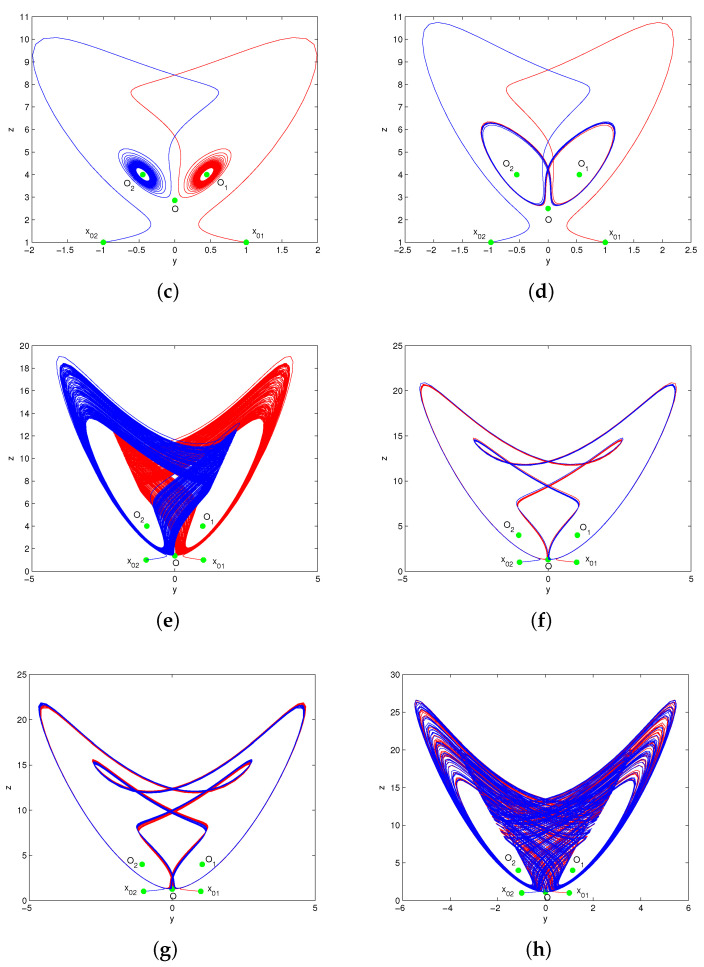
The phase portraits of system (1) with: (**a**) c=0.8; (**b**) c=1.2; (**c**) c=1.4; (**d**) c=1.6; (**e**) c=2.9; (**f**) c=3.1; (**g**) c=3.2; (**h**) c=3.6.

Consider the dynamic evolution of system (1) with respect to parameter *c* under the given parameter conditions a=2,b=8,k=4. The bifurcation diagrams of system (1) versus *c* ∈ (0, 6) are shown in Figure 4a, where the red color branch and blue color branch are yielded from initial values $x_{01}=\left(1,1,1\right),x_{02}=\left(-1,-1,1\right)$, respectively. The overlapped regions of the red color and blue color branches indicate that the trajectories of $x_{01},x_{02}$ eventually tend to the same attractor, while the separated regions indicate that the trajectories of $x_{01},x_{02}$ tend to different attractors. Figure 4b is the Lyapunov exponents of system (1) with initial value $x_{01}$. It shows that the system (1) experiences stable state, periodic state, chaotic state with the variation of *c*. When c∈(0,1), system (1) is mono-stable as it has only one stable equilibrium. When c∈(1,1.396), system (1) performs bi-stability with respect to the existence of two stable equilibria. As *c* increases across the critical value $c_{0}$ = 1.396, system (1) occurs double Hopf bifurcation at the equilibria. When c∈(1.396,1.516), system (1) performs bi-periodicity. When c∈(1.516,2.257), system (1) changes into mono-periodic state. When c∈(2.821,3.096), system (1) yields two strange attractors from initial values $x_{01},x_{02}$. When *c* ∈(3.310,4.167)∪(4.370,5.324)∪(5.480,6), system (1) has only one chaotic attractor. Table 1 describes the attractors of system (1) with different values of *c*. The phase portraits in Figure 5 illustrate the existence of different types of attractors in system (1).

### 4.2. Dynamic Evolution with Parameter k

**Figure 6:**
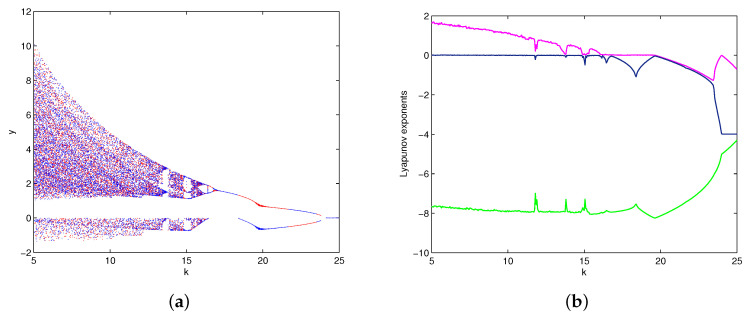
The bifurcation diagrams (**a**) and Lyapunov exponents (**b**) of system (1) versus k∈(5,25).

**Figure 7:**
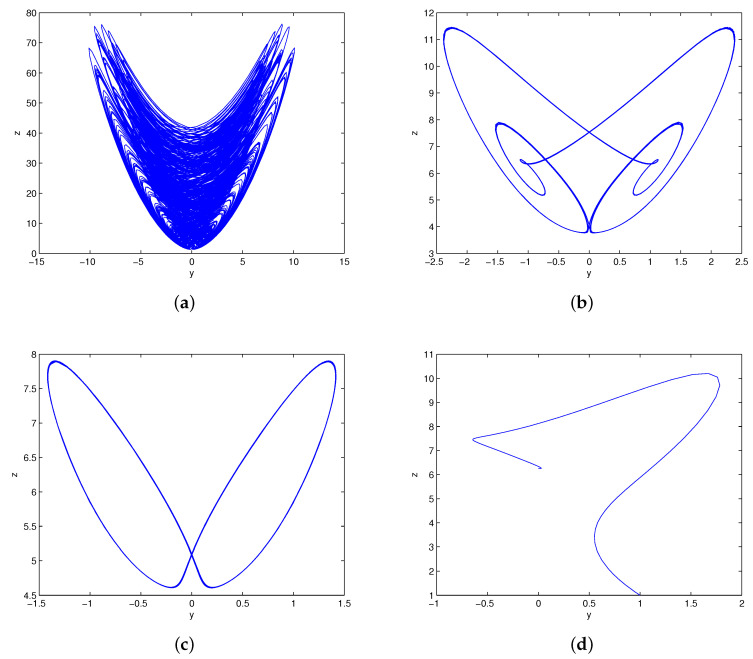
The phase portraits of system (1) with: (**a**) k=5; (**b**) k=15; (**c**) k=18; (**d**) k=25.

**Figure 8:**
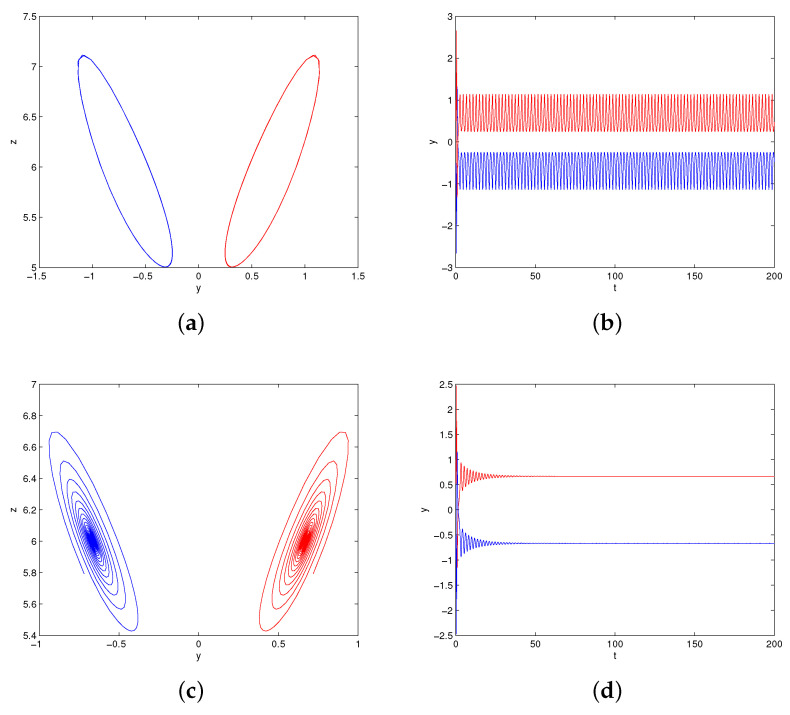
The coexisting attractors of system (1): (**a**) projections on x−y with k=19; (**b**) time series of *y* with k=19; (**c**) projections on x−y with k=20; (**d**) time series of *y* with k=20.

The bifurcation diagrams of system (1) for parameters (a,b,c)=(4,9,4), k∈(5,25) are shown in Figure 6a, where the red color branch and blue color branch are yielded from initial values $x_{01}$, $x_{02}$, respectively. Obviously, the state of system (1) changes from chaos to period and then to stable when parameter *k* increases from 5 to 25. It also can be illustrated by the Lyapunov exponents in Figure 6b. The maximum Lyapunov exponent is positive with c∈(5,13.6)∪(13.9,14.8)∪(15.4,15.9), negative with c∈(19.8,25), and equal to zero with c∈(13.7,13.8)∪(14.9,15.3)∪(16,19.7). For c=5,15,18,25, we can observe a strange attractor, a limit cycle, and a stable point of system (1), with their phase portraits are shown in Figure 7. For c=19,20, we can observe two coexisting periodic attractors and two coexisting point attractors of system (1), as shown in Figure 8.

## 5. Electronic Circuit Realization

There are many works that are related to chaos based applications in the literature [31,32,33,34,35,36]. Here, we will present the circuit realization of system (1) for realistically obtaining its chaotic attractors. The numerical simulation in Figure 5e displays two coexisting strange attractors in system (1) for (a,b,c,k)=(2,8,2.9,4) and initial conditions (±1,±1,1). For circuit realization of this state of system (1), we need to refrain from saturation of circuit elements, and the effective way to achieve this goal is to reduce the voltage values of the circuit via scaling the variables of system (1). In the process of scaling, we assume (X,Y,Z)=(x,y,z/2) and then the scaled system is obtained as

(8) $$\left\{\begin{array}{c} \dot{X}=aX-2YZ,\\ \dot{Y}=-bY+2XZ,\\ \dot{Z}=-cZ+XYZ+\frac{k}{2}. \end{array}\right.$$

**Figure 9:**
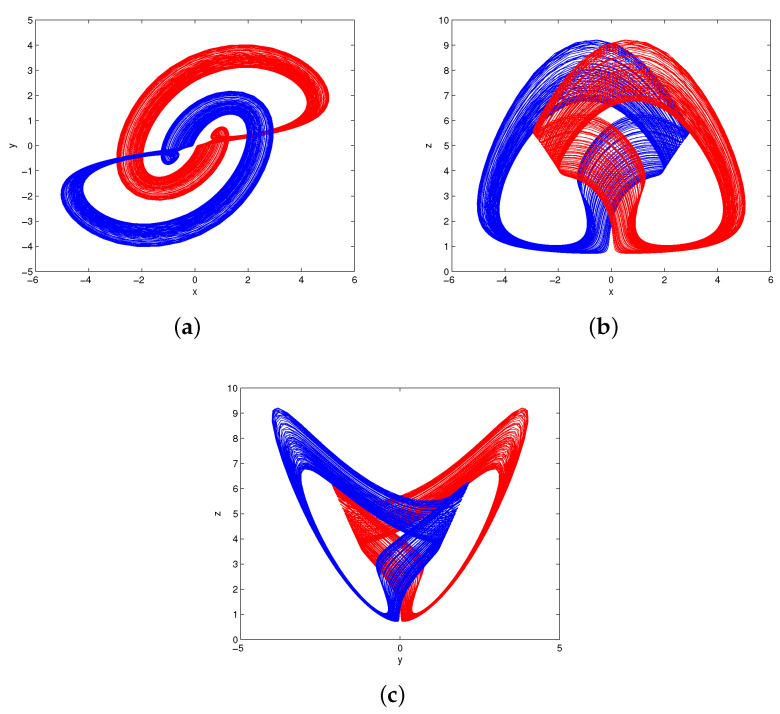
The phase portraits of the scaled system (8) for (a,b,c,k)=(2,8,2.9,4): (**a**) x−y; (**b**) x−z; (**c**) y−z.

Figure 9 gives the new phase portraits of the scaled system (8) for (a,b,c,k)=(2,8,2.9,4). Evidently, the scaling process does not cause fundamental changes to the system (1), but just limits the variables to a smaller region $\Omega =\{\left(x,y,z\right)\left|x,y\in (-5,5),\right.z\in \left(0,10\right)\}$.

**Figure 10:**
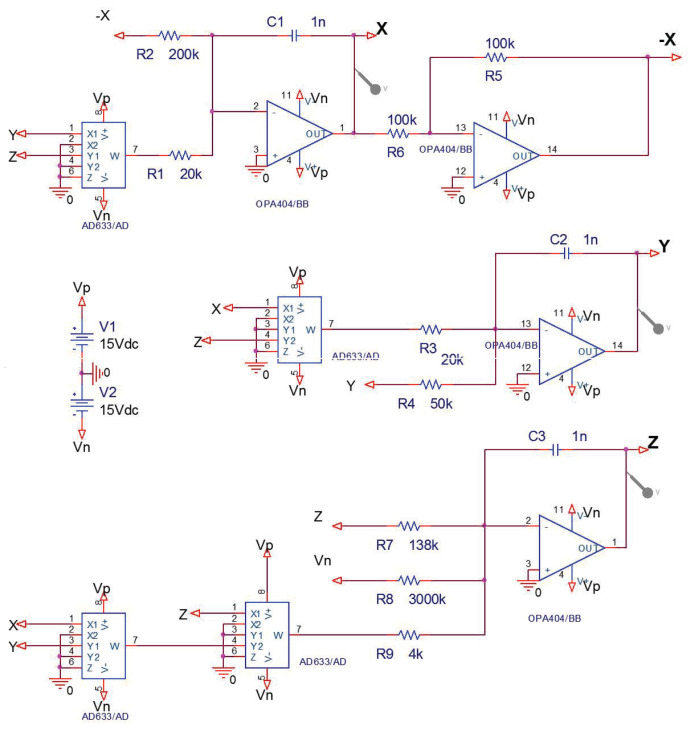
The circuit diagram of system (8).

**Figure 11:**
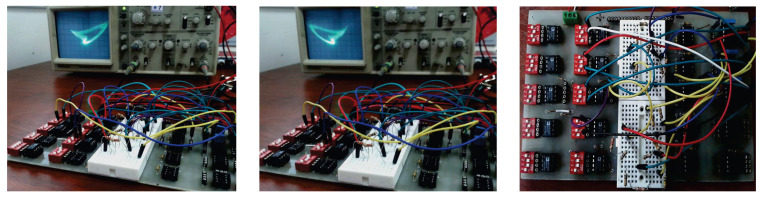
The experimental circuit of system (8).

**Figure 12:**
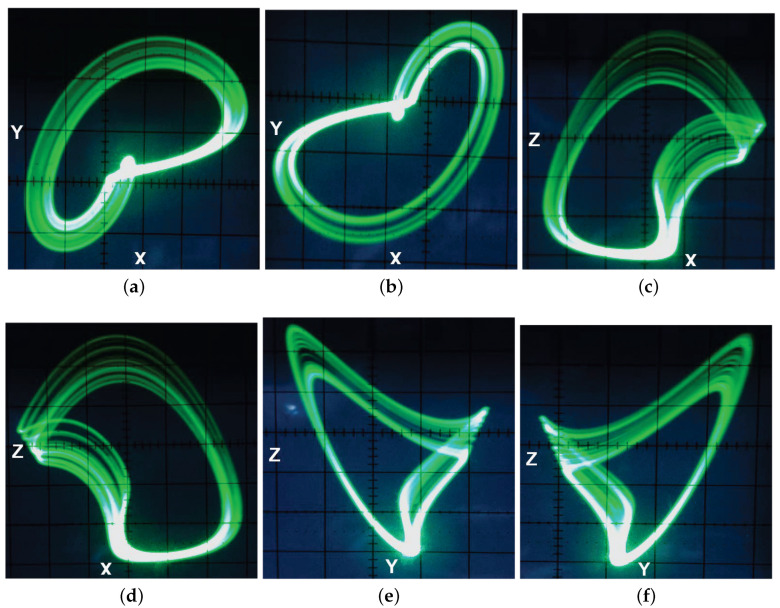
The phase portraits of two coexisting attractors of system (8) on the oscilloscope for (a,b,c,k)=(2,8,2.9,4): (**a,b**) x−y; (**c,d**) x−z; (**e,f**) y−z.

The circuit diagram of system (8) raised by the OrCAD-PSpice programme (OrCAD 16.6, OrCAD~company, Hillsboro, OR, USA) is presented in Figure 10. It has three input (or output) signals with respect to the variables X,Y,Z, and the operations between signals realized via the basic electronic materials including resistors, capacitor, TL081 operational amplifiers (op-amps), and AD633 multipliers. By fixing R1=R3=20 KΩ, R2=200 KΩ, R4=50 KΩ, R5=R6=100 KΩ, R7=138 KΩ, R8=3000 KΩ, R9=4 KΩ, C1=C2=C3=1 nF, Vn=−15 V, Vp=15 V and executing the circuit on electronic card shown in Figure 11, we can obtain the outputs of circuit in the oscilloscope. The oscilloscope graphics in Figure 12 show good consistency with the numerical simulations in Figure 9. Hence, we can come to a conclusion that the coexisting chaotic attractors in system (1) are physically obtained.

## 6. S-Box Design and Its Performance Analysis

This section aims to raise a new chaotic S-Box algorithm by applying system (1). In the algorithm design, first random number generation is performed and then S-Box is produced. The S-Box generation algorithm pseudo code is shown in Algorithm 1. For establishing the S-Box production algorithm, we first input the parameters (a,b,c,k)=(2,8,2.9,4) and initial value $\left(x_{0},y_{0},z_{0}\right)=\;\left(-1,-1,1\right)$ of the system and then the float number outputs are produced. In order to generate more random outputs in the analysis of the chaotic system, we select an appropriate step interval Δh and used it as a sample value. More random sequences are obtained by setting the appropriate step interval Δh=0.000001. System (1) is solved by using the RK4 algorithm with the initial conditions and the specified sampling value, and time series are obtained. In our designed chaotic S-Box algorithm, the outputs of *y*, *z* phases of system (1) are used. Float number values (32 bits) obtained from these phases are converted to a binary system. By taking 8 bits from the low significance parts (LSB) of the 32-bit number sequences generated from both phases, these values are XORed. The obtained new 8-bit value is converted to a decimal number. This value is discarded if the decimal number was previously generated and included in the S-Box, if not produced before, it is added to the S-Box. In this way, this process continues until the distinct 256 values (between 0 and 255) are obtained on the S-Box. The generated S-Box is shown in Table 2.

**Algorithm 1** The S-Box generation algorithm pseudo code.
 1:**Start**; 2:Inputting parameters and initial value of the system; 3:Sampling with step interval Δh; 4:*i* = 1, S-Box = []; 5:**while** (i<257) **do** 6:    Solving system with RK4 algorithm and obtaining time series (y,z); 7:    Convert float to binary number; 8:    Take LSB-8 bit value from RNG y⊕z phase; 9:    Convert binary to decimal number (8 bit)10:    **if** (Is there decimal value in S-Box = yes) **then**11:        Go step 6.12:    **else**(Is there decimal value in S-Box = no)13:        Sbox[*i*] ← decimal value;14:        *i*++;15:    **end**16:**end**17:S-Box ← reshape(Sbox,16,16);18:Ready to use 16×16 chaos-based S-Box;19:**End.**


In order to determine that the produced S-Boxes are robust and strong against attack, some performance tests are applied. We mainly focus on these tests: nonlinearity, outputs’ bit independence criterion (BIC), strict avalanche criterion (SAC), and differential approach probability (DP). In addition, the comparisons of the performance between this new S-Box and the existing chaotic S-Box proposed by Chen [37], Khan [38], Wang [39], Ozkaynak [40], Jakimoski [41], Hussain [42], Tang [43] are presented in Table 3.

Nonlinearity is regarded to be the most core part of all the performance tests. The nonlinearity values of the S-Box yielded by system (1) are obtained as 104, 106, 104, 104, 108, 104, 110 and 104. Accordingly, its average value, minimum value and maximum value are computed as 105, 104 and 110. By comparing the nonlinearity of other S-Box shown in Table 3, we can claim that the new S-Box is better than others in some measure.

SAC is put forward by Webster et al. [44]. Generally speaking, the establishment of SAC implies a possibility that half of each output bit will be changed with the change of a single bit. Table 3 tells the average, minimum and maximum SAC values of the new S-Box as 0.5014,0.3906,0.5937. Evidently, the average value of the new S-Box is close to the ideal value 0.5. BIC is also an important criterion found by Webster et al. [44]. It can partially measure the security of cryptosystems. The set of vectors generated by reversing one bit of the open text is tested to be independent of all the pairs of avalanche variables. While the relation between avalanches is measured, variable pairs are necessary to calculate the correlation value [45]. BIC-SAC and BIC-Nonlinearity values are calculated when the BIC value is calculated. When the values in Table 3 are examined, the BIC-SAC values are calculated as follows: average value 0.5028, minimum value 0.4394 and maximum value 0.5312. The average value is almost equal to the optimum value 0.5.

DP is another performance index for testing the S-Box, which is established by Biham et al. [46]. In this analysis, the XOR distribution balance between the input and output bits of the S-Box is determined. The very close probability of XOR distribution between input and output bits often indicates the ability to resist the differential attack of the S-Box. The low DP value suggests that the S-Box is more resistant to attack. The minimum and maximum DP values of the new S-Box are determined as 4.0 and 10. From Table 3, we know that the DP value of the new S-Box is the same as the S-Boxes presented by Tang and Ozkaynak.

After testing the performance of the new S-Box by using some important indices and comparing with other S-Boxes, we can determine that the new S-Box generated by system (1) has better performance than other S-Boxes. Thus, it will be more suitable for attack resistant and strong encryption.

## 7. Conclusions

A special chaotic system with multiple attractors was studied in this letter. The complex dynamic behaviors of the system were mainly presented by numerical simulations. Bifurcation diagrams and phase portraits indicated that the system exhibits a pair of point attractors, a pair of periodic attractors, and a pair of strange attractors with the variation of system parameters. In addition, an electronic circuit was designed for realizing the chaotic attractors of the system. Moreover, a new S-Box was generated by applying the chaotic system, and the performance evaluation and comparison of the S-Box were presented. It showed that the new S-Box has better performance than some existing S-Boxes. Actually, the study of chaotic system with multiple attractors is of recent interest. More important issues corresponding to this topic will be addressed in our future paper.

## Figures and Tables

**Table 1:** Attractors of system (1) with different values of *c*.

Value of *c*	Equilibrium Point	Type of Attractor	Figure
c=0.8	$\begin{array}{c} \mathrm{Stable}\;\mathrm{point}:\;(0,0,5) \end{array}$	A point attractor	Figure 5a
c=1.2	$\begin{array}{c} \mathrm{Saddle}\;\mathrm{node}:\;(0,0,3.3333)\\ \mathrm{Stable}\;\mathrm{point}:\;(\pm 0.6325,\pm 0.3162,4) \end{array}$	A pair of point attractors	Figure 5b
c=1.4	$\begin{array}{c} \mathrm{Saddle}\;\mathrm{node}:\;(0,0,2.8571)\\ \mathrm{Saddle}\;\mathrm{focus}:\;(\pm 0.8944,\pm 0.4472,4) \end{array}$	A pair of limit cycles	Figure 5c
c=1.6	$\begin{array}{c} \mathrm{Saddle}\;\mathrm{node}:\;(0,0,2.5)\\ \mathrm{Saddle}\;\mathrm{focus}:\;(\pm 1.0954,\pm 0.5477,4) \end{array}$	A symmetric limit cycle	Figure 5d
c=2.9	$\begin{array}{c} \mathrm{Saddle}\;\mathrm{node}:\;(0,0,1.3793)\\ \mathrm{Saddle}\;\mathrm{focus}:\;(\pm 1.9494,\pm 0.9748,4) \end{array}$	A pair of strange attractors	Figure 5e
c=3.1	$\begin{array}{c} \mathrm{Saddle}\;\mathrm{node}:\;(0,0,1.2903)\\ \mathrm{Saddle}\;\mathrm{focus}:\;(\pm 2.0494,\pm 1.0247,4) \end{array}$	A pair of limit cycles	Figure 5f
c=3.2	$\begin{array}{c} \mathrm{Saddle}\;\mathrm{node}:\;(0,0,1.25)\\ \mathrm{Saddle}\;\mathrm{focus}:\;(\pm 2.0976,\pm 1.0488,4) \end{array}$	A symmetric limit cycle	Figure 5g
c=3.6	$\begin{array}{c} \mathrm{Saddle}\;\mathrm{node}:\;(0,0,1.1111)\\ \mathrm{Saddle}\;\mathrm{focus}:\;(\pm 2.2804,\pm 1.1402,4) \end{array}$	A butterfly strange attractor	Figure 5h

**Table 2:** The chaotic S-Box of system (1).

199	30	5	41	38	140	230	139	66	0	11	195	76	204	54	23
254	198	50	108	231	92	87	182	217	28	56	253	219	232	215	49
102	151	68	86	176	248	12	32	126	249	141	154	82	138	174	165
145	62	115	150	201	104	170	148	78	97	192	247	252	96	211	153
45	98	40	91	109	113	196	107	209	83	144	120	191	75	242	208
175	246	100	181	85	70	197	136	235	210	93	216	71	105	162	149
88	240	31	238	42	171	90	73	112	243	255	128	239	121	26	34
25	226	59	244	135	142	53	36	146	157	117	124	116	10	205	60
173	29	2	72	203	3	214	224	127	241	143	74	6	156	122	61
110	8	1	233	79	51	77	47	236	222	185	152	180	15	103	234
206	227	169	202	137	221	177	179	163	52	245	67	89	80	220	7
237	183	17	4	101	37	39	57	178	194	58	69	213	147	18	228
46	35	225	84	14	125	95	134	129	63	99	55	106	161	218	27
250	21	13	24	207	193	48	184	189	114	111	167	16	160	188	123
155	132	158	130	118	166	164	168	33	159	223	64	44	81	190	172
212	20	229	186	65	251	133	22	131	43	119	94	19	9	187	200

**Table 3:** The comparison of different chaotic S-Boxes (BIC: bit independence criterion; SAC: strict avalanche criterion; DP: differential approach probability).

S-Box	Nonlinearity			BIC-SAC	BIC	SAC			DP
	Min	Avg	Max		Nonlinearity	Min	Avg	Max	
Proposed S-Box	104	105	110	0.5028	102.75	0.3906	0.5014	0.5937	10
Chen [37]	100	103	106	0.5024	103.1	0.4218	0.5000	0.6093	14
Khan [38]	96	103	106	0.5010	100.3	0.3906	0.5039	0.6250	12
Wang [39]	102	104	106	0.5070	103.8	0.4850	0.5072	0.5150	12
Ozkaynak [40]	100	103.2	106	0.5009	103.7	0.4218	0.5048	0.5938	10
Jakimoski [41]	98	103.2	108	0.5031	104.2	0.3761	0.5058	0.5975	12
Hussain [42]	102	105.2	108	0.5053	104.2	0.4080	0.5050	0.5894	12
Tang [43]	99	103.4	106	0.4995	103.3	0.4140	0.4987	0.6015	10
